# Development and initial validation of the cannabis-related psychosis risk literacy scale (CPRL): a multinational psychometric study

**DOI:** 10.1186/s12888-024-05727-x

**Published:** 2024-04-19

**Authors:** Feten Fekih-Romdhane, Amthal Alhuwailah, Hanaa Ahmed Mohamed Shuwiekh, Manel Stambouli, Abir Hakiri, Majda Cheour, Alexandre Andrade Loch, Souheil Hallit

**Affiliations:** 1 Faculty of Medicine of Tunis, Tunis Al Manar University, Tunis, Tunisia; 2 The Tunisian Center of Early Intervention in Psychosis, Department of Psychiatry Ibn Omrane, Razi Hospital, Tunis, Tunisia; 3 Department of Psychology, Kuwait University, Kuwait, Kuwait; 4 Department of Psychology, Fayoum University, Fayoum, Egypt; 5 Badr University in Cairo (BUC), Cairo, Egypt; 6 Laboratorio de Neurociencias (LIM 27), Instituto de Psiquiatria, Hospital das Clinicas HCFMUSP, Faculdade de Medicina, Universidade de Sao Paulo, Sao Paulo, SP, BR Brazil; 7 Instituto Nacional de Biomarcadores em Neuropsiquiatria (INBION), Conselho Nacional de Desenvolvimento Cientifico e Tecnológico, Sao Paulo, Brazil; 8 Psychology Department, College of Humanities, Effat University, 21478 Jeddah, Saudi Arabia; 9 Applied Science Research Center, Applied Science Private University, Amman, Jordan; 10 School of Medicine and Medical Sciences, Souheil Hallit, Holy Spirit University of Kaslik, Jounieh, P.O. Box 446, Lebanon

**Keywords:** Cannabis-related psychosis risk, Literacy, Psychosis, Cannabis, Scale development, Psychometric evaluation

## Abstract

**Background:**

Public education efforts to address and reduce potential harms from cannabis use in Arab countries are either slow or inexistent, and do not follow the steadily increasing trends of cannabis use in Arab youth. Several decades of research on substance use, it can be suggested that being aware of, and knowing about, psychosis risk related to cannabis can at least limit the consumption of the substance. Motivated by a lack of measures specifically designed to measure literacy about cannabis-related psychosis risk in younger populations, and based on an extensive literature review, we aimed to create and validate a new self-report scale to assess the construct, the Cannabis-related Psychosis Risk Literacy Scale (CPRL), in the Arabic language.

**Method:**

A cross-sectional study was carried-out during the period from September 2022 to June 2023, enrolling 1855 university students (mean age of 23.26 ± 4.96, 75.6% females) from three Arab countries (Egypt, Kuwait and Tunisia).

**Results:**

Starting from an initial pool of 20 items, both Exploratory Factor Analysis and Confirmatory Factor Analysis suggested that the remaining 8 items loaded into a single factor. The scale demonstrated good internal consistency, with both McDonald omega and Cronbach’s alpha values exceeding 0.7 (omega = 0.85 / alpha = 0.85). The CPRL showed measurement invariance across gender and country at the configural, metric, and scalar levels. Concurrent validity of the CPRL was established by correlations with less favourable attitudes towards cannabis (*r* = −.14; *p* <.001). In addition, higher literacy levels were found in students who never used cannabis compared to lifetime users (4.18 ± 1.55 vs. 3.44 ± 1.20, *t*(1853) = 8.152, *p* <.001).

**Conclusion:**

The newly developed CPRL scale offers a valid and reliable instrument for assessing and better understanding literacy about cannabis-related psychosis risk among Arabic-speaking young adults. We believe that this new scale is suitable as a screening tool of literacy, as an instrument for measuring the effect of public education interventions aimed at promoting cannabis-related psychosis risk literacy among young people, and as a research tool to facilitate future studies on the topic with a wider application.

**Supplementary Information:**

The online version contains supplementary material available at 10.1186/s12888-024-05727-x.

## Introduction

According to the World Health Organization and the American Psychiatric Association [1, 2], psychosis is conceptualized and defined as the presence of hallucinations without insight, delusions, or both. In the Diagnostic and Statistical Manual of Mental Disorders, Fifth Edition (DSM-5), psychosis is the defining feature of schizophrenia spectrum disorders (i.e., schizophrenia, schizoaffective disorder, brief psychotic disorder, schizophreniform disorder, and delusional disorder) [3]. Schizophrenia is, therefore, considered one of several psychotic disorders that exist on a spectrum of psychopathology [3]. Schizophrenia spectrum disorders are severe chronic diseases that entail extensive societal and health costs [4–6]. The aetiology of schizophrenia spectrum disorders is known to be multifactorial, involving a wide range of genetic and environmental risk factors [7]. One of the most consistently replicated environmental risk factors for psychosis is cannabis use [8]. An extensive amount of epidemiological, clinical and experimental studies have been devoted to demonstrate the causal link between cannabis use and subsequent development of psychotic symptoms or a schizophrenia spectrum disorder [9–13]. A succession of systematic reviews and meta-analyses of these data has demonstrated cannabis use as a risk factor in the development of psychosis later in life [11, 14–16]. For instance, Moore et al. [11] revealed that individuals who had ever used cannabis had 1.41-fold increased risk of any psychotic outcome. Semple et al. [16] concluded that cannabis use was associated with a 2.9-fold increase in schizophrenia or schizophrenia-like psychotic illness. A multicentre case-control study found an estimated proportion of first-episode psychosis attributable to high-potency cannabis use of 12.2% across 11 European sites, rising to 30.3% in London and 50.3% in Amsterdam [17]. Hjorthøj et al. [18] demonstrated that there has been a fourfold increase in the population-attributable risk fraction (from 2% prior to 1995 to 8% after 2010) for cannabis use disorder in schizophrenia in Denmark, mirroring the rising in use and potency of cannabis during the same period.

The role of cannabis use in the development of psychosis is complex [19], and appear to be moderated by some specific factors. These factors include the potency of the cannabis, the frequency of use, the age of first use, as well as certain genetic influences [9, 20–22]. Recent studies demonstrated the importance of genetic factors that render some individuals more prone to potential effects of cannabis [9, 23, 24]. Therefore, genetic predisposition is likely to play a determinant role in the risk cannabis use poses for the development of psychosis [9]. In this line, the polygenic risk score for schizophrenia (reflecting predisposition to psychosis) was consistently demonstrated to be related to increased use of cannabis [25, 26]. Previous Mendelian randomization studies [27, 28] showed causal pathways between schizophrenia risk and cannabis initiation/use. Overall, the literature available suggests that genetic predisposition to schizophrenia significantly accounts for variance in cannabis use [21]. In addition, a large body of evidence has shown that regular and frequent patterns of cannabis use are implicated in the multifactorial interplay toward psychosis development [8, 11, 29–31]. An umbrella review encompassing four meta-analyses indicated that cannabis use and the risk of psychosis are linked in a dose-response fashion [32]. Furthermore, there is clear evidence that psychosis-inducing effects of cannabis are further increased by the use of high-potency (skunk) cannabis, i.e. with greater levels of delta9-tetrahydrocannabinol (THC) [17, 31, 33, 34]. Laboratory studies confirmed that administering THC to healthy individuals has proven to induce new onset of transient psychotic symptoms [35–38]. Besides, THC-users were shown to display more severe positive psychotic symptoms and worse global functioning [39]. Synthetic cannabinoids are also demonstrated to produce psychoactive and physiological effects similar to THC, but with greater intensity, including psychosis [40]. Findings from meta-analyses support that individuals with a lower age of onset of cannabis use are at greater risk of psychosis [41–43]. Accordingly, a recent comprehensive review recommended delaying cannabis use initiation until after late adolescence as a strategy to reduce cannabis-related health harms [44]. There is solid evidence that cannabis use in adolescence and emerging adulthood has potential harms for the developing brain [45], and is linked to range of negative cognitive consequences [46–48]. Long-term cannabis users show specific cognitive deficits (such as IQ decline memory and attention problems, poor learning and processing speed) and smaller hippocampal volume in midlife [49]. Subsequently, cannabis may be a risk factor for psychosis even in less predisposed individuals in early stages of the disease [50]. Cannabis use has also a negative impact on social functioning and disease m outcomes in patients with schizophrenia spectrum disorders [51], is shown to influence disorganized symptoms [52] and to provoke an earlier transition to psychosis in those at a clinical high risk (CHR) for psychosis [53, 54]. Finally, cannabis has a differential effect on the Duration of Untreated Psychosis (DUP), as it can either lead to a rapid onset of psychotic symptoms (necessitating a rapid intervention), or result in a longer DUP due to several reasons (such as unwillingness to disclose cannabis use, self-medication, confounding psychotic symptoms by cannabis intoxication) [55].

In this sense, in a recent review authors stressed the “need for awareness campaigns to inform young people about the risks of psychosis associated with the use of cannabis” [32]. Likewise, Gage et al. [56] reviewed epidemiologic evidence from longitudinal research on causal relationship between cannabis exposure and psychosis, and concluded that there is “strong enough evidence to warrant a public health message that cannabis use can increase the risk of psychotic disorders”. Later, Murray et al. [57] supported this statement by pointing that, since no animal model for psychosis exits to date, “it is not sensible to wait for absolute proof that cannabis is a component cause of psychosis”. Therefore, cannabis has been recognized as “the risk factor for psychosis which is most readily open” to a universal primary prevention approach [57]. Evidence from other areas of medicine shows that avoidance of known and controllable risk factors in the entire population generates more public benefits and greater changes than efforts solely and intensively focusing on the affected individual. One effective and fruitful approach for psychosis prevention would be avoiding exposure to cannabis use as a well-established risk-increasing factor for psychosis. In this regard, Murray et al. [57] called for drawing inspiration from the great success of national campaigns against tobacco smoking which have led to remarkable decreases in smoking-related diseases, and advocated “public health campaigns to educate young people about the harms of regular use of high potency cannabis”.

Several decades of research on substance use provided insight into how enhanced health knowledge and perceived riskiness can positively influence substance use rates and indicators of substance-related adverse outcomes [58–62]. Being informed and aware of the non-normative nature of substance use and the misperceptions surrounding peer norms for such behaviours may help reduce young people’s tendency and intention to become users [63, 64]. In this regard, school-based programs (such as “Unplugged” [65]), have been widely implemented in several countries across Europe, and provided evidence for effectiveness in reducing cannabis use at short term. In addition, a systematic review and meta-analysis revealed that digital prevention and treatment interventions, mostly performed in Western countries (i.e. USA, Australia, and Canada), showed significant reduction effects on cannabis use that were maintained at follow-ups of up to 12 months [66]. However, a cross-country literature review reported that public health interventions (such as awareness-raising campaigns) are lacking in low-to-middle income countries, which choose to resort instead to legal prohibitions as a core strategy to combat cannabis use [67].

The Canadian Pediatric Society called for routine screening and motivational interviewing for cannabis use among youth using validated tools, in an effort to help them reflect on their behaviour and reduce related harm [68]. Despite the abundance of studies on the effects of knowledge or perceptions of risks on substance use in general (e.g [60, 61, 69])..,, the interplay between health knowledge, perceived risk, and cannabis use in particular is still largely understudied and misunderstood [70, 71]. In November 2022, the Canadian Centre on Substance Use and Addiction (CCSA) emphasized the existence of poor literacy and widespread misinformation about cannabis among Canadian youth, which limits their ability to make “informed choices” about their “cannabis health” ([ [72], p 5–7). For example, a previous study surveyed a sample of Canadian youth and young adults (*N* = 870; of whom a third reported being exposed to public health messages on cannabis) about the most important negative psychological effects associated with cannabis use using self-developed questions; it has been found that one in ten (10.9%) perceived “no risk” of harm for cannabis users’ mental health, and that the least frequently cited psychological concerns were hallucinations (3.7%), paranoia (3.4%), schizophrenia (1.1%), and psychosis (1.1%) [69]. A first major step toward enhancing public awareness regarding risks associated with cannabis use is to develop a psychometrically sound measure to assess literacy of cannabis-related psychosis risk. To answer the previous multiple calls for more public education efforts about cannabis use in youth [73], and those for universal primary prevention efforts aimed at preventing cannabis-related psychosis [56, 57], the present study sought to design and validate a new measure of cannabis-related psychosis risk literacy for use among adolescents and young adults.

### Rationale

Most Arab countries have gone through major political, social, and economic transformations over the past decade, that were associated with marked increases in cannabis availability and consumption [74]. For instance, the national 2021-Mediterranean School Survey Project on Alcohol and Other Drugs (MedSPAD) revealed that around 8% of Tunisian high school adolescents aged 16–18 reported lifetime cannabis use, 26.1% had close friends that use cannabis, and 16.2% perceived cannabis as easily accessible [75]. Other studies showed that 9% of Moroccan adolescents aged 16 years [76], 11% Kuwaiti male university students [77] and 12.3% of Lebanese male and female university students [78] reported lifetime cannabis use. Cannabis use is likely to be underreported in Arab countries and its prevalence rates may be largely underestimated because of its religious and legal prohibition Indeed, the use and possession of cannabis is strictly illegal in Arab countries (except Lebanon) [79]. For example, Tunisia strictly prohibits the sale and trafficking of cannabis, with cannabis use or possession being punishable by prison terms of one to five years and a monetary fine. Overall, prevalence estimates of cannabis use among Arab youth are high, as adolescents and young adults are at a challenging period of life where substantial brain development and critical life transitions occur. Adolescence and young adulthood is also a peak age for development of schizophrenia spectrum disorders [80]. At this age, early cannabis initiation is likely to progress to regular, chronic use, as patterns of cannabis consumption have a tendency to escalate over time in youth [81, 82]. On the other hand, public education efforts to address and reduce potential harms from cannabis use in Arab countries are either slow or inexistent, and do not follow the steadily increasing trends of cannabis use in Arab youth. Based on evidence that being aware of, and knowing about, psychosis risk related to cannabis can at least limit the consumption of the substance, making available a reliable and valid self-report measure for cannabis-related psychosis risk literacy may hopefully foster education initiatives based on harm reduction approaches, and provide new avenues for prevention in psychosis.

Motivated by a lack of measures specifically designed to measure literacy about cannabis-related psychosis risk in younger populations, and based on an in-depth, extensive and comprehensive literature review, we aimed to create and validate a new self-report scale to assess the construct, the Cannabis-related Psychosis Risk Literacy Scale (CPRL), in the Arabic language. More specifically, we sought to: (a) to explore the factorial structure of the CPRL in a sample of Arabic-speaking university students from three Arab countries (i.e. Egypt, Kuwait, Tunisia) using exploratory and confirmative factorial analysis techniques; (b) to establish measurement invariance of the CPRL between gender and countries; (C) to measure the reliability of the CPRL by Cronbach’s alpha and McDonald omega coefficients (internal consistency); (d) to examine the concurrent validity of the CPRL, by comparing and correlating its scores to lifetime cannabis use and attitudes toward cannabis.

## Methods

### Participants and procedure

All data were collected via a Google Form link, between March and June 2023. The research team contacted university students they knew. Those who accepted to participate were asked to forward the link to other students—snowball sampling technique. Inclusion criteria for participation included being of a resident and citizen of one of the three Arab countries (Egypt, Kuwait, Tunisia), aged over 18 years. An introductory paragraph was included at the beginning of the link explaining the objectives of the study, while assuring participants about confidentiality and anonymity of their responses. After providing digital informed consent, participants were asked to complete the instruments described above, which were presented in a pre-randomised order to control for order effects. Participants completed the survey voluntarily and without remuneration. The study protocol was approved by the ethics committee of Razi Hospital, Manouba, Tunisia.

#### Minimal sample size calculation

A sample between 100 and 200 participants was needed for the exploratory factor analysis based on 5–10 participants per scale’s item [83], whereas a sample between 60 and 400 participants was needed for the confirmatory factor analysis based on a previous study that suggested a minimum sample ranging from 3 to 20 times the number of the scale’s variables [84].

### Measures

Participants were asked to provide their age, gender (male/female), country of origin, Marital status (single/married/divorced/widowed), Living arrangement (alone/with partner or family members/with friends), Residency (rural/urban), and personal psychiatric history (yes/no). The socioeconomic status of participants was assessed by computing the household crowding index (i.e. the total number of people living in the household divided by the number of rooms in the dwelling, excluding bathrooms and kitchens) [85]. In addition, participants responded to the following measures in their Arabic versions.

#### Cannabis Use

Participants were asked to rate their cannabis use over the last six months on a five-point scale: 0 = Never, 1 = Monthly or less, 2 = 2–4 times a month, 3 = 2–3 times a week, and 4 = 4 or more times a week. This item was derived from the CUDIT-R [86]. Additionally, data on lifetime cannabis use (Yes/No) was collected.

#### The cannabis-related psychosis risk literacy scale (CPRL)

The development of the CPRL was conducted following different steps from Item generation to psychometric properties assessments. An extensive review of relevant literature and previous measures of mental health literacy (e.g [87–89]).., was performed to generate an initial pool of items. The item pool was ensured to be a rich source that is relevant to the content of interest. As such, the research team designed an initial questionnaire containing 20 items (see Appendix 1) and covering the following components: (a) knowledge and beliefs regarding symptoms induced by cannabis (e.g., “Hearing voices that do not actually exist can be a sign of cannabis use”), (b) knowledge and beliefs about mechanisms of the cannabis-psychosis relationship (e.g., “The genetic factor increases the risk of psychosis in cannabis users”), (c) pathways of the association between cannabis use and psychosis (e.g., “Stopping cannabis use can lead to a decline in psychotic symptoms and an improvement in functioning”), (d) knowledge and beliefs of professional help and treatment options (e.g., “The best way to deal with the symptoms of psychosis in a cannabis user is to deal with them on their own”). Items are measured as “true”, “I don’t know” and “false”. Each correctly answered item is scored one point, whereas any wrongly answered item is assigned a zero score. To the “I do not know” answer a zero score is also assigned. An expert panel (comprising three colleagues who are experts in clinical psychology and psychiatry) reviewed the items for conciseness and clarity. All items were sent out to 30 university students who were asked to write down their own interpretation of items while reading them.

#### The attitude about cannabis use scale

This scale has been developed and validated in the Arabic language [90]. It is composed of 14 items reflecting either favourable (e.g., “People have a good time when they use cannabis” or “The benefits of using cannabis outweigh the harms and risks associated with its use”) or unfavourable (e.g., “Cannabis use is a problem in our community” or “You would be concerned if a friend or family is using cannabis”) attitudes towards cannabis. Each item is scored on a five-point Likert-type scale, varying from 1 = strongly agree to 5 = strongly disagree. A total score is calculated by summing the ratings for all the 14 items, with higher scores indicating more favourable attitudes about cannabis use. The original Arabic scale yielded good psychometric properties among university students [90]. In the present study, a good Cronbach’s alpha value was found α = 0.838.

### Data Analysis

We used FACTOR 12.04.01 [91] to perform the Exploratory Factor Analysis (EFA) and to calculate reliability coefficients. Finally, we used the SPSS AMOS v.28 program to carry out the Confirmatory Factor Analysis (CFA).

To examine the internal structure of the test, we randomly divided the sample into two subsamples. We carried out an EFA in the first subsample, made up of 33% (1/3) of the total sample (604 subjects), and a CFA in the second subsample (1251 subjects). To check that the data was suitable for EFA we used KMO and Bartlett’s statistic. A preliminary analysis of the items was conducted using the Measure of Sampling Adequacy (MSA) at the item level [92], and (b) the Anti-Image Correlation (CAI) [93]. The MSA is a standardized index ranging from 0 to 1, with values below 0.50 considered unacceptable and leading to item elimination [92]. On the other hand, the Expected Residual correlation direct Change (EREC) index was used to assess the residual correlation between two items after removing the influence of all definable common factors in the dataset, hence, they should all be approximately 0. Item pairs with high shared correlation are referred to as doublets [93]. It is recommended to especially remove items that appear repeatedly in different doublets [94]. The exploratory factor analysis was carried out with a polychoric correlation matrix given the ordinal nature of the variables and the high number of items with kurtosis and skewness values greater than|1 [94, 95]. The method of estimation was Unweighted Least Squares (ULS), following the guidelines in the current literature [96]. We determined the number of factors using the Optimal Implementation of Parallel Analysis (PA) procedure [97, 98].

Subsequently, in order to confirm the dimensionality indicated by the EFA, we performed a CFA with the second subsample. The method of estimation used was Maximum likelihood estimates. The indices of fit were *Comparative Fit Index* (CFI), Tucker-Lewis Index (TLI), Standardized Root Mean Square Residual (SRMR) and *Root Mean Square Error of Approximation* (RMSEA). A good fit was observed if CFI and TLI > 0.90, SRMR < 0.05 and RMSEA < 0.08 [99]. Multivariate normality was not verified at first (Bollen-Stine bootstrap *p* =.002); therefore, we performed non-parametric bootstrapping procedure (available in AMOS).

**Gender invariance.** To examine gender and country invariance of CPRL scores, we conducted multi-group CFA [100] using the total sample. Measurement invariance was assessed at the configural, metric, and scalar levels [101]. We accepted ΔCFI ≤ 0.010 and ΔRMSEA ≤ 0.015 or ΔSRMR ≤ 0.010 as evidence of invariance [100, 102]. We aimed to test for gender and countries differences on latent CPRL scores using an independent-samples *t*-test and ANOVA tests respectively only if scalar or partial scalar invariance were established.

We used Cronbach’s alpha coefficient and McDonald’s ω coefficient to examine reliability. The CPRL score was considered normally distributed since the skewness and kurtosis values varied between ± 1.96. For the bivariate analysis, the Student t and ANOVA tests were used to compare two and three or more means, whereas the Pearson correlation test explored the correlation between CPRL and other scores. We used Bonferroni’s correction for multiple testing; the adjusted p value (= 0.005) was calculated by dividing 0.05 by the total number of variables being tested (= 10). A linear regression was conducted afterwards taking the CPRL score as the dependent variable and all factors that showed a *p* <.005 as independent variables. *P* <.05 was deemed statistically significant.

## Results

Characteristics of the sample.

A total of 1855 participants from 3 countries filled the survey, with a mean age of 23.26 ± 4.96 and 75.6% females. Other characteristics of the sample are found in Table 1.

**Table 1:** Characteristics of the sample (*n* = 1855)

	n (%)
Country	
Egypt	558 (30.1%)
Kuwait	821 (44.3%)
Tunisia	476 (25.7%)
Gender	
Male	453 (24.4%)
Female	1402 (75.6%)
Marital Status	
Single/Separated/Divorced	1557 (83.9%)
Married	298 (16.1%)
Living arrangement	
Alone	206 (11.1%)
With partner/family members	1588 (85.6%)
With friends	61 (3.3%)
Residency	
Urban	1485 (80.1%)
Rural	370 (19.9%)
Lifetime cannabis use	
No	1642 (88.5%)
Yes	213 (11.5%)
Cannabis use over the last six months	
Never	1813 (97.7%)
Monthly or less	29 (1.6%)
2–4 times a month	11 (0.6%)
2–3 times a week	2 (0.1%)
4 or more times a week	0 (0%)
Age (years)	23.26 ± 4.96
Household crowding index	1.40 ± 0.72
Attitudes about Cannabis Use scores	47.64 ± 9.41
CPRL scores	4.09 ± 1.53

CPRL: Cannabis-related Psychosis Risk Literacy Scale

Exploratory factor analysis.

We first performed an EFA on the first subsample of 150 subjects. The relevance of the items was analyzed using the MSA index; 12 items were suggested to be removed (items 1, 2, 3, 4, 5, 6, 9, 13, 15, 16, 17, 18) because of values lower than 0.50. Another factor analysis was then conducted after removal of those items. The suitability of the data was confirmed via a good KMO value (= 0.884) and a Bartlett’s test of sphericity *p* value < 0.001. Results indicated an adequate fit to a unidimensional structure supported by the GFI (GFI = 0.99) being greater than 0.95, the explained variance of 62%, the RMSEA (RMSEA = 0.02) less than 0.05, the UniCo (UniCo = 0.974) index greater than 0.95, the I-ECV (= 0.875) greater than 0.85 and MIREAL (MIREAL = 0.250) lower than 0.30. Parallel analysis indicated that a one-factor model would best fit the data. Composite reliability of scores was adequate for the total score (ω = 0.83 / α = 0.83).

CFA was done on the second subsample. The fit indices of the unidimensional model of the CL scores was acceptable for all indices, RMSEA = 0.093 (90% CI 0.082, 0.104), SRMR = 0.047, CFI = 0.908, TLI = 0.934. The modification index between items 19 and 20 was high (= 75.01); consequently, a correlation was added between the two residuals; the fit indices improved as follows: RMSEA = 0.076 (90% CI 0.066, 0.088), SRMR = 0.036, CFI = 0.958, TLI = 0.938. The factor loadings from the EFA and the standardised estimates of factor loadings from the CFA were all adequate (Table 2). Composite reliability of scores was adequate for the total score (ω = 0.85 / α = 0.85).

**Table 2:** Factor Loadings of the CPRL items from the Exploratory Factor Analysis (EFA) in the first subsample and Confirmatory Factor Analysis (CFA) in the second subsample

Items	Percentage of correct responses	Percentage of “I don’t know” responses	EFA	CFA
1. Cannabis causes structural changes in the brain of users (T)	1148 (48.6%)	563 (23.8%)	0.77	0.70
2. All people who use cannabis will develop psychological symptoms (F)	530 (22.4%)	821 (34.8%)	0.78	0.61
3. Individuals who use cannabis are at greater risk of developing diseases such as schizophrenia (T)	1088 (46.1%)	713 (30.2%)	0.83	0.75
4. The genetic factor increases the risk of psychosis in cannabis users (T)	1162 (49.2%)	624 (26.4%)	0.74	0.63
5. The risk of developing psychotic symptoms increases when consuming high-potency cannabis (i.e. with high THC levels) (T)	1082 (45.8%)	466 (19.7%)	0.87	0.76
6. Stopping cannabis use can lead to a decline in psychotic symptoms and an improvement in functioning (T)	1072 (45.4%)	654 (27.7%)	0.78	0.65
7. The best way to deal with the symptoms of psychosis in a cannabis user is to deal with them on their own (F)	655 (27.7%)	538 (22.8%)	0.57	0.40
8. Religious practices and prayer help prevent or control the symptoms of psychosis that may appear in cannabis users (F)	963 (40.8%)	687 (29.1%)	0.66	0.55

CPRL: Cannabis-related Psychosis Risk Literacy Scale. F: False; T: True

### Measurement invariance in the second subsample

The results shown in Table 3 suggest measurement invariance across gender and countries. A higher CPRL score was significantly found in females compared to males (4.19 ± 1.55 vs. 3.80 ± 1.44, *t*(1249) = -4.09, *p* <.001). Moreover, a one-way analysis of variance showed that the mean CPRL score was highest in Egypt (*M* = 4.63, *SD* = 1.47), followed by Tunisia (*M* = 4.07, *SD* = 1.69) and Kuwait (*M* = 3.76, *SD* = 1.36). The difference was significant for the whole trend, *F*(2,1248) = 37.59, *p* <.001, and between countries taken two by two (*p* <.05 for all comparisons).

**Table 3:** Measurement Invariance across gender and country in the second subsample

Model	CFI	RMSEA	SRMR	Model Comparison	ΔCFI	ΔRMSEA	ΔSRMR	
Model 1: Gender								
Males	0.953	0.083	0.039					
Females	0.958	0.073	0.036					
Configural invariance	0.956	0.053	0.039					
Metric invariance	0.955	0.050	0.051	Configural vs. metric	0.001	0.003	0.012	
Scalar invariance	0.946	0.051	0.056	Metric vs. scalar	0.009	0.001	0.005	
Model 2: Countries								
Egypt	0.978	0.056	0.031					
Kuwait	0.965	0.073	0.033					
Tunisia	0.882	0.085	0.057					
Configural invariance	0.959	0.042	0.031					
Metric invariance	0.943	0.044	0.035	Configural vs. metric	0.016	0.002	0.004	
Scalar invariance	0.899	0.053	0.035	Metric vs. scalar	0.044	0.009	< 0.001	

CFI = Comparative fit index; RMSEA = Steiger-Lind root mean square error of approximation; SRMR = Standardised root mean square residual

### Bivariate analysis (in the total sample)

A higher mean CPRL score was found in participants from Egypt compared to Kuwait and Tunisia, in females compared to males, in those living with a friend, in those living in rural areas and in those who used cannabis during their lifetime or in the last 6 months (Table 4). In addition, more favourable attitudes towards cannabis (*r* = −.14; *p* <.001) was significantly associated with lower CPRL scores. It is of note that age (*r* = −.03; *p* =.164) and household crowding index (*r* =.04; *p* =.102) were not significantly associated with Cannabis-related Psychosis Risk Literacy.

**Table 4:** Bivariate analysis of factors associated with Cannabis-related Psychosis Risk Literacy

	Mean ± SD	t / F	df / df1,df2	p
Country		55.94	2, 1852	**< 0.001**
Egypt	4.62 ± 1.46			
Kuwait	3.76 ± 1.37			
Tunisia	4.05 ± 1.69			
Gender		-4.23	1853	**< 0.001**
Male	3.84 ± 1.46			
Female	4.18 ± 1.54			
Marital Status		1.42	1853	0.157
Single/Separated/Divorced	4.12 ± 1.54			
Married	3.98 ± 1.50			
Living arrangement		10.97	2, 1852	**< 0.001**
Alone	3.70 ± 1.49			
With partner/ family members	4.13 ± 1.52			
With friends	4.62 ± 1.74			
Residency		-3.00	1853	**0.003**
Urban	4.04 ± 1.55			
Rural	4.31 ± 1.44			
Lifetime cannabis use		8.15	1853	**< 0.001**
No	4.18 ± 1.55			
Yes	3.44 ± 1.20			
Cannabis use over the last six months		5.74	1853	**< 0.001**
No	4.12 ± 1.53			
Yes	3.19 ± 1.02			

Numbers in bold indicate significant p values (*p* <.005) after Bonferroni correction for multiple testing

### Multivariable analysis (in the total sample)

Females compared to males (Beta = 0.21) had significantly higher CPRL scores, whereas Kuwaiti participants compared to Egyptian ones (Beta = -1.01), living in rural areas compared to urban (Beta = − 0.34), ever used cannabis (Beta = -1.27) and having used cannabis in the last 6 months (Beta = -1.44) were significantly associated with lower CPRL scores (Table 5).

**Table 5:** Multivariable analysis of factors associated with Cannabis-related Psychosis Risk Literacy

	Unstandardized Beta	Standardized Beta	p	95% CI
Sex (females vs. males*)	0.21	0.06	**0.014**	0.04; 0.37
Country (Kuwait vs. Egypt*)	-1.01	− 0.33	**< 0.001**	-1.20; − 0.82
Country (Tunisia vs. Egypt*)	− 0.03	− 0.01	0.817	− 0.28; 0.22
Living arrangement (with partner/family vs. alone*)	− 0.19	− 0.04	0.175	− 0.47; 0.09
Living arrangement (with a friend vs. alone*)	0.001	0.001	0.997	− 0.46; 0.46
Residency (rural vs. urban*)	− 0.34	− 0.09	**0.001**	− 0.54; − 0.14
Lifetime cannabis use (yes vs. no*)	-1.27	− 0.26	**< 0.001**	-1.59; − 0.94
Cannabis use in the last 6 months (yes vs. no*)	-1.44	− 0.14	**< 0.001**	-1.94; − 0.95
Cannabis attitudes	− 0.01	− 0.04	0.123	− 0.01; 0.002

*Reference group; numbers in bold indicate significant *p* values

## Discussion

Several researchers claimed that evidence on the causal effect of cannabis exposure on psychosis is sufficiently solid to merit attention and action. They have, therefore, raised the urgent need to start addressing this primary prevention issue in psychosis, and start large-scale campaigns to educate young people about the harms of cannabis use [32, 56, 57]. To contribute in advancing the field, this study represents the first attempt to develop and psychometrically test a measurement instrument, the CPRL, to evaluate literacy about cannabis-related psychosis risk among young people. After completing the validity and reliability phases, the CPRL consisted of eight items which loaded into one single factor. The factor structure was invariant across gender and country groups. Therefore, the CPRL appears to be an easy-to-use and cost-effective self-administered scale that can now be utilized to evaluate knowledge of signs, symptoms, mechanisms, pathways, help-seeking and treatment options of cannabis-related psychosis among Arabic-speaking youth. We believe that this new scale is suitable as a screening tool of literacy, as an instrument for measuring the effect of public education interventions aimed at promoting cannabis-related psychosis risk literacy among young people, and as a research tool to facilitate future studies on the topic with a wider application.

This study adopted both EFA and CFA techniques to examine the structure of the CPRL, as recommended in the literature [103]. This approach enabled a first examination of the factor structure of the CPRL via EFA without modelling limitations in a first subsample of 150 Arabic-speaking university students. Then, as a next step, to cross-validate the EFA-derived model of CPRL scores in a second subsample via CFA. Results provided support to a single-factor structure of CPRL scores across both split-half subsamples with good model fit and adequate factor loadings. Furthermore, the scale demonstrated good internal consistency, with both McDonald omega and Cronbach’s alpha values exceeding 0.7 (omega = 0.85 / alpha = 0.85) [104]. The CPRL showed measurement invariance across gender and country at the configural, metric, and scalar levels. In other words, the unidimensional model of the scale was equivalent between male and female students, and functioned similarly for Arabic-speaking young adults from three different Arab countries and cultures (one Gulf country, i.e. Kuwait, one Middle East, i.e. Egypt, and one North-African/Maghrebian, i.e. Tunisia). These findings advocate that CPRL items are interpreted in a conceptually similar way by respondents representing specific gender and country groups of students. This suggests that any noted differences in CPRL scores across gender or country groups are engendered by genuine differences in levels of knowledge about cannabis-related psychosis risk rather than a differential functionality of the scale for these groups. As such, the CPRL appears to be a fair and precise measure for use in future studies to make between-groups comparisons of cannabis-related psychosis risk literacy. Finally, correlation analyses showed that better literacy levels were positively correlated with less favourable attitudes about cannabis use. This means that students who had a better knowledge about the risk of psychosis related to cannabis use endorsed more negative attitudes towards cannabis. In addition, lower literacy levels were found in students who ever used cannabis in their lifetime compared to non-users. These results support concurrent validity of the CPRL, and lend additional support to previous literature stipulating that enhanced knowledge and perceived riskiness may affect attitudes and behaviours toward substance use [58–64].

Our study revealed that only approximately half of the students recognized as true that “Cannabis causes structural changes in the brain of users” and that “Individuals who use cannabis are at greater risk of developing diseases such as schizophrenia”. In addition, 40.8% endorsed that the statement “religious practices and prayer help prevent or control the symptoms of psychosis that may appear in cannabis users” was true, and 27.7% approved the statement that “the best way to deal with the symptoms of psychosis in a cannabis user is to deal with them on their own”. These findings are in line with a previous study on cannabis health knowledge, known risks, and risk perception among young Canadians, which showed that around 11% perceived no risk of harm from using cannabis [69]. When participants were asked to list up to five most important negative health effects from cannabis use, only 1.1-3.7% cited psychotic symptoms and disorders as possible effects [69]. A substantial proportion of our students thought that cannabis users with psychosis would better turn to religious practices and prayer or deal with their symptoms on their own. A large multi-country study showed that a significant proportion of Arab individuals tend to endorse religious/supernatural causations of mental illness, and to prefer informal sources of help such as traditional religious healers and Cleric [105]. Another previous study in Arab young people from the general population found that greater religiosity was associated with higher levels of stigma, and that the effect of religiosity on stigma can be modifiable through improving literacy [106]. Altogether, the present study provides a first overview of knowledge on psychosis risk subsequent to cannabis use among Arab young adults, and suggests that much remains to be done in terms of information and education for the general public in Arab countries.

### Limitations and implications for future research

Some limitations need to be discussed before conclusions can be drawn. First, the study may be subject to social desirability response bias because of the potential sensitivity of the topic covered. Second, predictive validity could not be tested because of the cross-sectional nature of the study. Third, the snowball sampling technique followed to recruit participants predisposes us to a selection bias. Fourth, participants were recruited online, with an overrepresentation of females, which may limit the representativeness of the sample. This is, however, a typical issue with convenience samples in online surveys [107]. Fifth, other cannabis-related information, such as the type and potency of cannabis used was not gathered in the context of the present study. This is due to the fact that, in Arab countries, illegal market cannabis is often obtained from an unknown source. As such, the cannabis consumed locally is of unknown type and potency levels. Sixth, other important psychometric properties of the CPRL (such as test-retest reliability) were not examined. Finally, the present study involved Arabic-speaking participants from three different Arab countries. Therefore, psychometric characteristics of the CPRL still need to be verified in other languages and cultural backgrounds, such as English and the Western world.

## Conclusion

The newly developed CPRL scale offers a valid and reliable instrument to assess and better understand literacy about cannabis-related psychosis risk among Arabic-speaking young adults. This understanding is necessary to prevent both cannabis use and possible development of subsequent psychosis. The CPRL can be easily administered and demonstrated good psychometric properties. Use of The CPRL scale is expected to allow for an easy and effective identification of young people at-risk for psychosis who may benefit from further education or support. In addition, the CPRL could be used as a monitoring tool to ensure the efficiency of programs designed to improve cannabis-related psychosis risk literacy in both clinical and non-clinical settings. Future psychometric studies of the CPRL in other environments and cultures are warranted to further confirm the psychometric qualities of the scale for different youth populations.

### Electronic supplementary material

Below is the link to the electronic supplementary material.

Appendix 1

## Data Availability

The datasets generated and/or analyzed during the current study are not publicly available due to restrictions from the ethics committee but are available from the corresponding author on reasonable request.
