# Supplementary material for: Development and initial validation of the cannabis-related psychosis risk literacy scale (CPRL): a multinational psychometric study
Source: BMC Psychiatry. 2024 Apr 19;24:298. doi: 10.1186/s12888-024-05727-x (PMC11027227; doi:10.1186/s12888-024-05727-x)
Supplement: Supplementary file 1 — Appendix 1 [file 12888_2024_5727_MOESM1_ESM.docx]

**Appendix 1.** The initial pool of the Cannabis-related Psychosis Risk Literacy Scale’s items in English.

|  | **True** | **False** | **I do not know** |
| --- | --- | --- | --- |
| 1. Cannabis use improves social life, communication with others, and increases the ability to feel pleasure. **†** |  | * |  |
| 1. Hearing voices that do not actually exist (e.g., noises, music, buzzing, or whispering) can be a sign of cannabis use. **†** | * |  |  |
| 1. Cannabis users generally suffer from thought disorders (e.g., accelerated thinking, flight of ideas, slurred speech, jumping from one idea to another with no apparent connection, difficulty communicating a message). **†** | * |  |  |
| 1. Cannabis consumption helps improve concentration, attention and memory. **†** |  | * |  |
| 1. Cannabis can cause delusions (i.e., false or irrational beliefs, such as having superpowers, being watched or spied on). **†** | * |  |  |
| 1. Cannabis has a positive effect on the normal development of the nervous system. **†** |  | * |  |
| 1. Cannabis causes structural changes in the brain of users | * |  |  |
| 1. The genetic factor increases the risk of psychosis in cannabis users | * |  |  |
| 1. Tetrahydrocannabinol (known as THC) is the component of cannabis that causes the symptoms of psychosis. **†** | * |  |  |
| 1. The risk of developing psychotic symptoms increases when consuming high-potency cannabis (i.e. with high THC levels) | * |  |  |
| 1. Individuals who use cannabis are at greater risk of developing diseases such as schizophrenia | * |  |  |
| 1. All people who use cannabis will develop psychological symptoms |  | * |  |
| 1. People who use cannabis at an older age (e.g., 18 years old) are at greater risk of developing psychosis than those who use cannabis at an earlier age (e.g., 15 years old). **†** |  | * |  |
| 1. Stopping cannabis use can lead to a decline in psychotic symptoms and an improvement in functioning | * |  |  |
| 1. A single use of cannabis cannot cause psychosis. **†** |  | * |  |
| 1. When a cannabis user develops symptoms of psychosis, they should not tell anyone, and should instead wait until symptoms improve on their own. **†** |  | * |  |
| 1. Psychotherapy provided by a mental health professional can be effective in treating symptoms of psychosis in cannabis users. **†** | * |  |  |
| 1. Antipsychotic medications are effective in treating symptoms of psychosis in cannabis users. **†** | * |  |  |
| 1. Religious practices and prayer help prevent or control the symptoms of psychosis that may appear in cannabis users |  | * |  |
| 1. The best way to deal with the symptoms of psychosis in a cannabis user is to deal with them on their own |  | * |  |

**†** Items removed.
